# Symbiont specificity differs among green hydra strains

**DOI:** 10.1098/rsos.220789

**Published:** 2022-10-12

**Authors:** Ryo Miyokawa, Maki Hanada, Yumiko Togawa, Taichi Q. Itoh, Yoshitaka Kobayakawa, Junko Kusumi

**Affiliations:** ^1^ Graduate School of Integrated Science for Global Society, Kyushu University, 744 Moto-oka, Nishi-ku, Fukuoka 819-0395, Japan; ^2^ Graduate School of Systems Life Science, Kyushu University, 744 Moto-oka, Nishi-ku, Fukuoka 819-0395, Japan; ^3^ Faculty of Arts and Science, Kyushu University, 744 Moto-oka, Nishi-ku, Fukuoka 819-0395, Japan; ^4^ Department of Environmental Changes, Faculty of Social and Cultural Studies, Kyushu University, 744 Moto-oka, Nishi-ku, Fukuoka 819-0395, Japan; ^5^ Graduate School of Life and Environmental Sciences, University of Tsukuba, 1-1-1, Ten-noudai, Tsukuba, Ibaraki 305-8572, Japan

**Keywords:** green hydra, symbiosis, *Hydra viridissima*, *Chlorella*

## Abstract

The symbiotic hydra *Hydra viridissima* has a stable symbiotic relationship with the green alga *Chlorella*. This hydra appears to cospeciate with the symbiotic alga, and some strains are known to have strain-specific host/symbiont combinations. To investigate the mechanism of the specificity between host and symbiont, we explored the effect of the removal or exchange of symbionts in two distantly related *H. viridissima* strains (K10 and M9). In the K10 strain, severe morphological and behavioural changes were found in symbiont-removed and symbiont-exchanged polyps. Interestingly, both polyps showed a similar gene expression pattern. The gene ontology (GO) enrichment analysis revealed that the removal or exchange of symbionts caused the downregulation of genes involved in the electron transport chain and the upregulation of genes involved in translation in the K10 strain. On the other hand, symbiont-removed and symbiont-exchanged M9 polyps showed modest changes in their morphology and behaviour compared with the K10 strain. Furthermore, the patterns of the gene expression changes in the M9 strain were quite different between the symbiont-removed and symbiont-exchanged polyps. Our results suggested that the regulation of energy balance is one of the crucial mechanisms for maintaining symbiotic relationships in green hydra, and this mechanism differs between the strains.

## 1. Introduction

Symbiosis is a long-term, close and reciprocal biological interaction between multiple organisms [1], and it enables organisms to adapt to new niches and diversify their lifestyle [2,3]. Photosymbiosis, which is widespread across multiple animal taxa, is a particular case of animal–plant symbiosis. In photosymbiosis, hosts protect their symbionts from predators and environmental fluctuations, and the symbionts provide the hosts with nutrients derived from photosynthetic products [4]. The photosymbiotic systems in cnidaria (corals, sea anemones and hydras) are widely known to establish symbiotic relationships with zooxanthellae or green algae, and these symbioses have profound effects on the lifecycle and ecology of cnidarians [5–7].

Green hydra (*viridissima* species group) have a stable symbiosis with algae, typically *Chlorella* sp., in their endodermal epithelial cells, which are brightly greened by symbionts [8–11]. Green hydra have been studied for a long time as one of the most suitable systems for research on symbiosis [12–14]. Recently, whole-genome sequences of a green hydra strain and its symbiotic *Chlorella* have been determined (*Hydra viridissima* A99, [15,16]), enabling us to perform functional genomic analysis. Polyps of the green hydras harbour symbiotic chlorellae, which are enveloped in membranes called symbiosomes in endodermal cells. The hydras provide the symbiotic chlorellae with carbon and nitrogen sources for photosynthesis and receive maltose produced by photosynthetic reactions in return [5,17,18]. This symbiotic interaction allows the host hydra to increase the proliferation rate by budding and tolerance to starvation [19]. The molecular phylogenetic analysis of the hydra species indicated that the green hydra is a basal clade that diverged from other hydra species groups over 100 million years ago [20]. The green hydra seems to have acquired symbiosis with chlorellae at the early stage of its evolution because all green hydras have symbiotic relationships with chlorellae [21]. After the acquisition of symbionts, this species has diverged into multiple strains with large genetic distances [20,21]. As a result of long and tight symbiotic partnerships, the symbiotic *Chlorella* have evolved high dependence on their hosts; they cannot survive outside the host cell for a long time. The symbiotic chlorellae are transmitted vertically to offspring polyps by asexual proliferation by budding or sexual reproduction through algal migration into an egg [21,22]. In addition, some green hydra strains have evolved strong symbiont/host specificity.

The symbiont/host specificity was investigated by symbiont-exchange experiments in green hydra [15,23,24]. These studies indicated that some host hydras allow specific symbiotic chlorellae to be taken into the endoderm cells, and non-native symbionts can have negative impacts on the host's proliferation. For example, the symbiotic green hydra harbouring *Chlorella variabilis* NC64A, the symbiotic alga with *Paramecium bursaria*, shows a lower proliferation rate by budding than the hydra harbouring the native symbiotic *Chlorella* [15]. On the other hand, a few studies suggested that symbiont/host specificity in green hydra is not so strict. Kawaida *et al*. [21] exchanged symbiotic chlorellae among green hydra strains and successfully maintained some new combinations of symbiont/host partnerships. Symbiotic *Chlorella* of four diverged *viridissima* strains tested (M9, M8, K10 and J8) could increase in number in other apo-symbiotic strains (B5 and N11), and the new symbiotic relationships could last for at least a month. Furthermore, Rahat and Reich [25,26] reported that an apo-symbiotic polyp of the green hydra became infected artificially with free-living *Chlorella* species. They reported that not all *Chlorella* strains established a symbiotic relationship with hydras, and the *Chlorella* strains that were susceptible to infection had resistance to acidity. However, it remains unclear whether specificity in the combination between symbionts chlorellae and host hydra is stringent and what mechanisms act on the specificity.

This study analysed the host-symbiont specificity of the green hydra using two diverged *H. viridissima* strains (K10 and M9). First, we established green hydras harbouring non-native symbionts and then investigated their morphological changes, proliferation rates and gene expression patterns. The results showed that there were severe phenotypic changes in the K10 polyps when the symbionts were removed or exchanged, but those changes were modest in M9, and the overall gene expression pattern reflected the phenotypic differences between the strains.

## 2. Material and methods

### 2.1. *Hydra* strains and microinjection experiments

Experiments were carried out with *H. viridissima* strain K10 (Swiss strain) and strain M9 (Israel strain) (electronic supplementary material, figure S1*a*,*d*). The symbiotic strains K10 and M9 (denoted K10ori and M9ori) and the apo-symbiotic strains derived from the above strains were provided by the National Institute of Genetics (NIG) at Mishima, Japan (code nos. of the apo-symbiotic strains are B5 and N11, respectively). We labelled these apo-symbiotic hydras K10apo and M9apo, respectively (electronic supplementary material, figure S1*b*,*e*). K10apo and M9apo were used as hosts in the following microinjection experiments to create symbiont-exchanged hydras (electronic supplementary material, figure S1*g*) [21].

We labelled the two symbiont-exchanged hydras, the K10 polyp with the M9 chlorellae and the M9 polyp with the K10 chlorellae, K10exc and M9exc, respectively (electronic supplementary material, figure S1*c*,*f*,*g*). To determine if any damage occurred during the microinjection experiments, we checked the reproduction rate of the symbiont-restored hydras (K10res and M9res) produced by microinjection of native symbionts to the apo-symbiotic hydras.

All hydras used in this study were kept in plastic containers filled with hydra culture solution (HCS; 1 mM NaCl, 1 mM CaCl_2_, 0.1 mM KCl, 0.1 mM MgSO_4_, 1 mM tris-(hydroxmethy1)-aminomethane; pH 7.4, adjusted with HCl) at 20°C under 12 h light/12 h dark illumination cycles (84 µmol m^−2^ s^−1^ light intensity) and were fed newly hatched nauplii of *Artemia* sp. twice a week. The day after feeding, polyps were transferred into another container filled with fresh HCS.

### 2.2. Phenotypic measurements

#### 2.2.1. Proliferation rate of polyps

Six polyps for each experimental group (K10ori, M9ori, K10apo, M9apo, K10res, M9res, K10exc and M9exc) were used to estimate the proliferation rates. At the start of the observation, we placed one polyp per well in a six-well cell culture plate filled with 10 ml of HCS. We counted the number of polyps reproducing asexually by budding from one polyp for more than three weeks. The start date of proliferation was assumed to be the day that the first bud appeared. The doubling time was calculated with a polyp being 1 and a bud being 0.5.

The sexual reproduction ability of polyps was evaluated by egg production. We placed 10 polyps per well into a six-well cell culture plate filled with 10 ml HCS, and the number of eggs was recorded for 40 days with feeding every 3 days. To keep the number of polyps per well at 10 throughout the experiments, we removed eggs and new polyps generated by budding.

#### 2.2.2. Observation of endoderm epithelial cells

The dissected body column of a polyp without the head and foot was placed on a glass slide. After removal of the HCS, we gently added a drop (approx. 50 µl) of the hydra maceration solution (acetic acid : glycerin : DW = 1 : 1 : 13) onto the sample tissue. After 20 min of incubation, we tapped the sample tissue with a fine needle and dissociated it into single cells. Then, a coverslip was placed on the sample, which was observed with a Nomarski differential interference microscope (ECLIPSE Ni-U, Nikon, Tokyo).

#### 2.2.3. Measurement of the number of stenoteles per tentacle and predatory ability

The head (hypostome and tentacles) of the polyps was dissected and placed on a glass slide with a small amount of HCS. A coverslip was applied carefully to prevent the extended tentacles from overlapping each other. The sample between the glass slide and the coverslip was pressed appropriately, and then we counted all stenoteles in a tentacle under a Nomarski differential interference microscope. We counted the stenoteles from 8 to 14 polyps for each experimental group (K10ori, M9ori, K10apo, M9apo, K10exc and M9exc) and used the estimates per tentacle.

Next, we evaluated the predatory ability of tentacles. The polyp was transferred to a well of a 24-cell culture plate filled with 1 ml of HCS 3 days after the last feeding. One hydra polyp was placed in a well, and 10 nauplii of *Artemia* sp. were gently added. The polyps were left for 1 day to allow them to eat the prey until satiety. One day later, the number of remaining nauplii in each well was scored. We considered the number of nauplii missing after one day as the number of predations. Six polyps from each of the six experimental groups were tested simultaneously, and the same operation was performed six times.

### 2.3. Transcriptome analysis

#### 2.3.1. RNA extraction and RNA-seq analysis

We used six experimental groups (K10ori, M9ori, K10apo, M9apo, K10exc and M9exc) for the RNA-seq analysis. Total RNA was extracted from 30 intact polyps starved for three days using the acid guanidinium thiocyanate-phenol-chloroform (AGPC) method [27] and RNeasy Plus Mini Kit (QIAGEN, Hilden, Germany). Two biological replicates were prepared for each experimental group. The extracted RNA was treated with DNase I (Roche, Mannheim, Germany) to remove genomic DNA. The total RNA samples for differential gene expression analysis were sent to Novogene Co., Ltd (Beijing, China) for cDNA library construction and sequencing of 150 bp paired-end reads on an Illumina HiSeq 4000. Then, we sequenced the total RNA samples using 300 bp paired-end reads for de novo assembly. mRNA was extracted from the total RNA samples using the NEBNext Poly(A) mRNA Magnetic Isolation Module (New England Biolabs, Ipswich, MA). cDNA libraries were constructed using the TruSeq Stranded mRNA library prep (Illumina, San Diego, CA). These libraries were sequenced using Illumina MiSeq. The sequence data were deposited in the DDBJ/EMBL/Genbank under the accession numbers: SRR21134050–SRR21134067.

#### 2.3.2. De novo assembly and annotation of hydra contigs

Low-quality ends (QV < 30) and adapter sequences were trimmed, and short reads (<20 bp) were discarded for quality control using cutadapt 1.12 [28]. Any remaining rRNA sequences were filtered out using SortMeRNA 2 [29]. After quality filtering, we obtained 144 million read pairs for the K10 strain and 138 million for the M9 strain. De novo assembly [30] was performed on the reads generated from HiSeq and MiSeq using Trinity 2.3.2, and the contigs less than 200 bp were discarded. This process yielded 161 896 contigs in the K10 strain and 191 536 contigs in the M9 strain. Next, reads from HiSeq were mapped to the assembled contigs using salmon 0.14.1 [31]. The average mapping rates were 89.5% in the K10 strain and 89.2% in the M9 strain (median for the K10 strain: 89.6%; M9 strain: 89.1%).

Clustering of the assembled contigs and read counting were performed with Corset 1.08 [32], and we obtained 52 384 contigs for the K10 strain and 58 773 contigs for the M9 strain. A similarity search of the clustered contigs against UniProtKB was conducted using BLASTX 2.5.0 with an e-value cut-off of 1e-5 to remove sequences from the symbiotic chlorellae and annotate the contigs. For each contig, we calculated e-values against homologous protein sequences of Opisthokonta and Viridiplantae in UniProtKB and those of *H. vulgaris* in TrEMBL. Contigs whose e-values of the best hits against Opisthokonta and *Hydra* sequences were smaller than those against Viridiplantae were considered hydra contigs. These hydra contigs were annotated with UniProtKB Swiss-Prot, and the remaining unannotated contigs were annotated with entries from *H. vulgaris*, whose whole genome was sequenced and annotated [33] in UniProtKB TrEMBL to find hydra-specific genes. The number of contigs annotated by UniProtKB Swiss-Prot was 16 202 in the K10 strain and 15 430 in the M9 strain, and that annotated by *Hydra* entries in UniProtKB TrEMBL was 21 297 and 19 899, respectively. These annotated contigs were considered possible hydra-derived contigs, and the annotated contigs with counts per million mapped reads (CPM) greater than 0 were used for subsequent differential gene expression analyses. The assembled contig sequences and the clustering information are available at https://doi.org/10.5061/dryad.2fqz612rq.

To find orthologues between K10 and M9, protein sequences translated from the contigs were estimated using TransDecoder 5.5.0 (https://github.com/TransDecoder/TransDecoder), and an orthogroup, which is the set of genes that are descended from a single gene in the last common ancestor, was inferred by Orthofinder 2.2.7 [34] using those protein sequences and sequences from *H. vulgaris* in the RefSeq database.

#### 2.3.3. Differential gene expression analysis and gene ontology enrichment analysis

Differential gene expression analyses were performed between K10ori, K10apo and K10exc and between M9ori, M9apo and M9exc using edgeR [35]. We used the original symbiotic polyps as a control, and contigs with false discovery rate (FDR) < 0.05 were considered to be differentially expressed genes (DEGs).

Gene ontology (GO) enrichment analysis was performed using the DAVID Functional Annotation tool [36]. UniProt accession numbers of DEGs with any CPM were used as the inputs in the GO enrichment analysis. GO Direct was used among GO categories for the GO enrichment analysis. GO terms with Benjamini–Hochberg corrected *p*-values < 0.05 by Fisher's exact test were considered to be significantly enriched among the differentially expressed genes.

#### 2.3.4. Quantitative PCR analysis

For quantitative PCR (qPCR) analyses, RNA samples were prepared from 10 to 30 polyps for each experimental group using TRIzol Reagent (Thermo Fisher Scientific). After the treatment with DNase I (Roche, Mannheim, Germany), cDNAs were synthesized from total RNAs using Oligo(dT)12–18 Primer (Invitrogen) with SuperScript III (Thermo Fisher Scientific) according to the manufacturer's instructions. Real-time PCR was performed with Mx3000P (Agilent Technologies) using Brilliant III Ultra-Fast SYBR Green QPCR Master Mix (Agilent Technologies). The PCR conditions were 2 min at 94°C, followed by 35 cycles of 94°C for 30 s and 50°C for 60 s. Translation elongation factor 1*α* (EF1*α*) was used as the internal control for normalization. Data were averaged from three independent experiments.

## 3. Results

### 3.1. Exchange of symbiont chlorellae affected the proliferation of host polyps in the K10 strain

We estimated the doubling time (day) for each experimental group (table 1) based on the number of polyps and buds generated by asexual reproduction. In the K10 strain, there was a significant difference in the doubling time among the experimental groups (Kruskal–Wallis test, *p* = 0.0028). Notably, the average doubling times for K10exc and K10apo were significantly larger than that for K10ori (table 1, Steel's test, *p* < 0.001). K10res also showed a significantly larger doubling time than K10ori (Steel's test, *p* < 0.01), but the difference was small, suggesting that reinjection of native symbionts somewhat restored the proliferation efficiency by budding in the K10 polyp. For M9, there was no significant difference in the doubling time among the experimental groups (Kruskal–Wallis test, *p* = 0.69), but there were significant differences between M9exc and M9ori (Steel's test, *p* < 0.001) and between M9res and M9ori (Steel's test, *p* < 0.05). The results showed that removal or replacement of symbionts affected the asexual proliferation rate in the K10 and M9 polyps, and severe reduction occurred in K10apo and K10exc. Next, we evaluated sexual reproduction ability based on the number of eggs. We could not find any egg production in K10apo and K10exc, but the other strains could generate approximately 6–8 eggs for 40 days (sum of 10 polyps). These results showed that the asexual and sexual reproduction abilities of the K10 polyps were significantly affected by their symbiotic state, but these effects were modest in the M9 polyps.

**Table 1.** Asexual proliferation rates and egg production.

strain	experimental group	doubling time (day)		number of eggs^a^
		*n*	average ± SE	
K10	K10ori	6	4.45 ± 0.10	5
	K10apo	6	7.80 ± 1.23***	0
	K10exc	6	8.33 ± 1.51***	0
	K10res	6	5.32 ± 0.43**	8
M9	M9ori	6	4.29 ± 0.07	5
	M9apo	6	4.70 ± 0.27	8
	M9exc	6	4.87 ± 0.11***	8
	M9res	6	5.07 ± 0.29*	7

Notes: ^a^Eggs generated from 10 polyps within 40 days.

Significantly different from the original symbiotic polyps (K10ori or M9ori) (Steel's test, *** *p* < 0.001, ** *p* < 0.01, * *p* < 0.05).

### 3.2. Substantial morphological changes occurred in K10exc and K10apo

**Figure 1.**
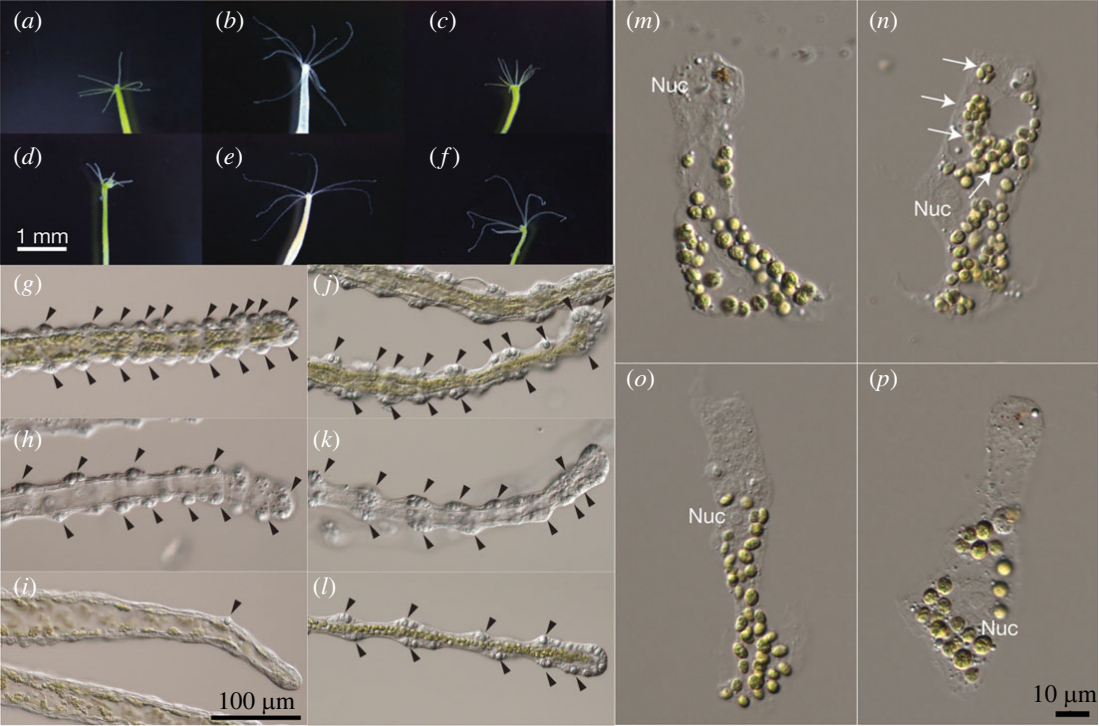
(*a*–*f*): Tentacles of K10ori (*a*), K10apo (*b*), K10exc (*c*), M9ori (*d*), M9apo (*e*) and M9exc (*f*). Original symbiotic hydras normally have 6–7 tentacles. Some of the K10apo and K10exc polyps have larger numbers of tentacles. (*g*–*l*): Stenoteles of K10ori (*g*), K10apo (*h*), K10exc (*i*), M9ori (*j*), M9apo (*k*) and M9exc (*l*). Black arrows indicate stenoteles. (*m*–*p*) Macerated endodermal epithelial cells of K10ori (*m*), K10exc (*n*), M9ori (*o*) and M9exc (*p*). The upper side of each photograph is the apical (gastric cavity) side, and the lower side is the basal (mesoglea) side of the cells. Aggregations of chlorellae are indicated by white arrows. Nuc: nucleus of the endodermal cell.

Tentacles are essential for hydra polyps to catch prey and move, and the original symbiotic polyps normally had approximately six tentacles (figure 1*a*–*f*, table 2). We found a significant difference in the number of tentacles among the experimental groups of the K10 strain (Kruskal–Wallis test, *p* < 0.001). K10apo had a significantly larger number of tentacles (9.33 ± 0.43) than K10ori (Steel's test, *p* < 0.001). Most of the K10apo polyps had more than 8 tentacles, and some had 11 tentacles. Although the difference was not significant, the K10exc polyps had a large variation in the number of tentacles, which ranged from 5 to 10. For the M9 strain, there was no significant difference among the experimental groups (table 2, Kruskal–Wallis test, *p* = 0.17).

**Table 2.** Morphological changes in tentacles.

strain	experimental group	number of tentacles		number of stenoteles		predation success	
		*n*	average ± SE	*n*	average ± SE	*n*	average ± SE
K10	K10ori	12	6.50 ± 0.26	8	104.1 ± 5.3	36	4.06 ± 0.38
	K10apo	12	9.33 ± 0.43***	8	61.4 ± 6.3***	36	5.42 ± 0.36*
	K10exc	12	7.25 ± 0.41	14	41.8 ± 11.0***	36	2.17 ± 0.42**
M9	M9ori	12	6.83 ± 0.17	7	227.7 ± 12.9	36	6.97 ± 0.19
	M9apo	12	6.50 ± 0.20	7	142.4 ± 22.0	36	5.25 ± 0.22***
	M9exc	12	6.42 ± 0.19	7	199.5 ± 16.2	36	7.08 ± 0.28

Significantly different from the original symbiotic polyps (K10ori or M9ori) (Steel's test, *** *p* < 0.001, ** *p* < 0.01, * *p* < 0.05).

In addition, we found that the number of stenoteles per tentacle differed among the experimental groups across both strains (figure 1*g*–*l*, table 2). K10ori had approximately 100 stenoteles per tentacle, while M9ori had approximately 200 stenoteles. The K10apo and M9apo polyps had approximately 40% fewer stenoteles than each original polyp, and the differences were significant in K10 (Steel's test, *p* < 0.001). For the symbiont-exchanged polyps, K10exc had a drastically reduced number of stenoteles compared with K10ori but had more stenoteles than K10apo (Steel's test, *p* < 0.001). On the other hand, M9exc showed a comparable number of stenoteles to M9ori.

We expected that the change in the number of tentacles and stenoteles may affect the predation efficiency of polyps, so we measured predation success per feeding (table 2). For the M9 strain, the difference in predation success among the polyps within an experimental group was not significant (ANOVA, d.f. = 5, *p* = 0.775), but there was a significant difference among the experimental groups (ANOVA, d.f. = 2, *p* < 0.01). The M9ori polyps preyed on 6.97 out of 10 artemia per feeding on average. The predation success of M9apo (5.25 per feeding) was slightly lower than that of M9ori (Steel's test, *p* < 0.001). There was no significant difference between M9ori and M9exc. In the K10 strain, the differences among the polyps within the experimental group were not significant (ANOVA, d.f. = 5, *p* = 0.206), but there was a significant difference among the experimental groups (ANOVA, d.f. = 2, *p* < 0.001). The K10ori polyps on average preyed on 4.06 artemia per feeding, which was less than M9ori. The lowest success in the K10 strain was found for K10exc (2.17 per feeding), which was significantly lower than that for K10ori (Steel's test, *p* < 0.01). There was a small but significant difference between K10ori and K10apo (Steel's test *p* < 0.05). Increasing the number of tentacles in K10apo may somewhat contribute to predation success. In both strains, the most severe reduction in predation success was observed in the polyps with the smallest number of stenoteles, suggesting that the predation efficiency somewhat correlates to the number of stenoteles.

Morphological changes occurred not only in the hosts but also in the symbionts (figure 1*m*–*p*). Each native symbiotic chlorella of the K10 polyp and that of the M9 polyp is normally wrapped with membrane in endodermal epithelial cells and forms a single symbiosome. Symbiosomes are normally located at the basal side (mesoglea side) of the cell and are separated from one another (figure 1*m*,*o*). However, such a well-ordered structure was impaired in K10exc. In the macerated endodermal cells of K10exc, some aggregations of chlorellae were found in a single symbiosome (figure 1*n*) but not in the others. In addition, chlorellae were located on the upper side (gastric lumen side) in the cells of K10exc. Morphological and behavioural abnormalities in the host and symbiont were predominantly found in K10exc, as shown by their reproduction abilities, which seem to be attributed to host–symbiont incompatibility.

### 3.3. Differential gene expression analysis

To identify the genes responsible for the difference in the symbiotic mechanism between the two strains, we compared gene expression patterns between the experimental groups for each strain.

**Figure 2.**
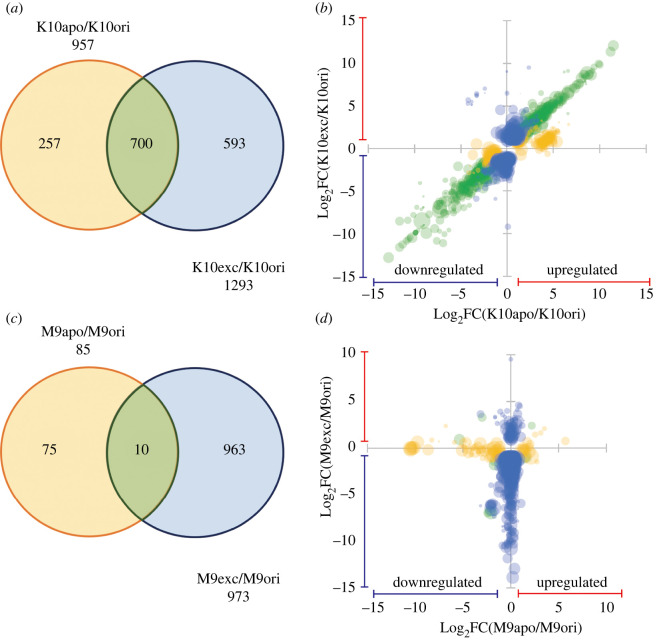
Gene expression changes in apo-symbiotic (K10apo, M9apo) and symbiont-exchanged (K10exc, M9exc) hydras compared with the original symbiotic hydras (K10ori, M9ori) (FDR < 0.05). (*a*): Venn diagram representing the number of DEGs found in the K10 strain. (*b*): Bubble chart of the fold changes of DEGs between K10apo and K10exc. (*c*): Venn diagram representing the number of DEGs found in the M9 strain. (*d*): Bubble chart of the fold changes of DEGs between K10apo and K10exc. The yellow colour represents the DEGs unique to the apo-symbiotic hydras (K10apo, M9apo), and the blue colour represents the DEGs unique to symbiont-exchanged hydras (K10exc, M9exc). Green bubbles represent DEGs significant in both the apo-symbiotic and symbiont-exchanged hydras.

Among the annotated contigs of the K10 strain, 957 and 1293 genes were significantly differentially expressed in the K10apo polyps and the K10exc polyps, respectively, compared with the original K10 polyps (figure 2*a*, FDR < 0.05). Of these, 700 genes were identified in both comparisons, 593 DEGs were unique to K10apo, and 257 DEGs were unique to K10exc. In addition, the fold changes of DEGs found in the K10apo polyps and the K10exc polyps showed a significant positive correlation (*R* = 0.84, *p* < 0.001, figure 2*b*). This result indicated that the K10apo and K10exc polyps had similar gene expression changes from the original polyps.

Next, we performed gene ontology enrichment analysis of the DEGs with UniProtKB Swiss-Prot annotation (table 3). GO terms enriched in upregulated and downregulated DEGs of K10apo were also significantly enriched in those of K10exc. Most of the enriched GO terms for upregulated DEGs in both K10apo and K10exc were responsible for translation, including ribosomal proteins, eukaryotic translation initiation factors and elongation factors (electronic supplementary material, table S1). The GO term ‘integral component of membrane’ was enriched in downregulated DEGs in both K10apo and K10exc, including cytochrome c oxidases, NADH-ubiquinone oxidoreductases and ammonium transporters (electronic supplementary material, table S2). Similar to those for ribosomal proteins, the genes for the products constituting the tricarboxylic acid (TCA) cycle and the electron transport chain, which are related to cellular metabolism, were upregulated in the K10apo polyps (electronic supplementary material, table S3). The downregulated DEGs unique to the K10exc polyps contained ion channels related to nematocyte discharge and factors promoting nematocyte differentiation (electronic supplementary material, table S4).

**Table 3.** Enriched GO terms of the DEGs found in the K10 strain.

category^a^	term	count^b^	pop hits^c^	fold enrichment^d^	*q* value^e^
*K10apo > K10ori*					
MF	**structural constituent of ribosome**	36	285	4.26	1.31 × 10^−10^
BP	**translation**	35	309	3.83	1.74 × 10^−8^
CC	**cytosolic large ribosomal subunit**	17	108	5.45	1.54 × 10^−5^
CC	**ribosome**	19	174	3.78	2.84 × 10^−4^
CC	**extracellular exosome**	59	1135	1.80	4.49 × 10^−4^
BP	**cytoplasmic translation**	11	68	5.47	0.012697
*K10apo < K10ori*					
MF	**cytochrome-c oxidase activity**	8	26	12.88	5.79 × 10^−4^
CC	i**ntegral component of membrane**	66	1962	1.52	0.035332
*K10exc > K10ori*					
MF	**structural constituent of ribosome**	57	285	5.15	1.31 × 10^−22^
BP	**translation**	56	309	4.78	4.80 × 10^−20^
CC	**ribosome**	32	174	4.91	6.02 × 10^−11^
CC	**cytosolic small ribosomal subunit**	20	62	8.62	6.19 × 10^−11^
CC	cytosolic large ribosomal subunit	25	108	6.18	8.09 × 10^−11^
CC	cytosolic ribosome	12	30	10.69	3.45 × 10^−7^
BP	**cytoplasmic translation**	15	68	5.82	9.47 × 10^−5^
CC	**extracellular exosome**	72	1135	1.69	2.81 × 10^−4^
BP	ribosomal large subunit assembly	10	34	7.76	0.001144
BP	ribosomal small subunit assembly	7	21	8.79	0.025099
*K10exc < K10ori*					
CC	**integral component of membrane**	119	1962	1.65	4.96 × 10^−7^
MF	**cytochrome-c oxidase activity**	12	26	11.77	5.75 × 10^−7^
CC	respiratory chain	12	41	7.94	2.34 × 10^−5^
CC	respiratory chain complex IV	7	10	19.00	4.17 × 10^−5^
BP	homophilic cell adhesion via plasma membrane adhesion molecules	11	40	7.60	0.001352
MF	calcium ion binding	32	340	2.40	0.001928

Notes: ^a^category of GO term, ^b^number of DEGs enriched in a certain term, ^c^the number of all genes enriched in a certain term, ^d^degree of over-representation of DEGs compared with the background, ^e^the *p*-value adjusted using the Benjamini-Hochberg procedure. Significantly enriched GO terms in both K10apo and K10exc are shown in bold face.

There were 85 and 973 significant DEGs in the M9apo polyps and M9exc polyps, respectively, compared with the original M9 polyps (figure 2*c*, FDR < 0.05). The number of significant DEGs found in the M9 strain was smaller than that in the K10 strain. Of these, only 10 DEGs were shared between the two above comparisons, and most of the significant DEGs were unique to either M9apo or M9exc. We found 75 DEGs unique to the M9apo polyps and 963 DEGs unique to the M9exc polyps. The fold changes of the DEGs in the M9apo and M9exc polyps had no correlation (*R* = −0.086, figure 2*d*), suggesting that the changes in gene expression pattern from the original symbiotic polyps were quite different between M9apo and M9exc.

GO enrichment analysis revealed that there were no significantly enriched GO terms for either upregulated or downregulated DEGs in the M9apo polyps. Removal of symbionts did not significantly change the gene expression pattern from the original symbiotic strain. However, the genes encoding ammonium transporters and ascorbate peroxidases, which are related to nutrient exchange between symbionts and hosts or active oxygen scavenging, were downregulated in the M9apo polyps (electronic supplementary material, table S5), suggesting that several genes have responded to symbiont removal. On the other hand, a few significantly enriched GO terms were found in the M9exc polyps (table 4). The GO term ‘voltage-gated calcium channel complex’ was the only significantly enriched term in the upregulated DEGs in the M9exc polyps, while five GO terms (e.g. ‘extracellular matrix’ and ‘calcium ion binding’) were enriched in the downregulated DEGs. The upregulated genes with the term ‘voltage-gated calcium channel complex’ were calcium channels, which are related to nematocyte discharge [37,38]. At the same time, polycystins and ion channels were downregulated in M9exc, although these genes are also related to nematocyte discharge (electronic supplementary material, table S6). In addition, the GO term ‘extracellular matrix’ contained collagens, which constitute mesoglea, and cadherins, which function in cell adhesion (electronic supplementary material, table S6). Similar downregulation of cadherin genes was previously reported in apo-symbiotic M9 polyps [19].

**Table 4.** Enriched GO terms in the DEGs found in the M9 strain.

category^a^	term	count^b^	pop hits^c^	fold enrichment^d^	*q* value^e^
M9exc > M9ori					
CC	voltage-gated calcium channel complex	4	23	26.92	0.039995
M9exc < M9ori					
CC	extracellular matrix	18	93	3.69	0.001915
CC	extracellular exosome	84	1024	1.56	0.003694
MF	calcium ion binding	40	343	2.12	0.005426
CC	proteinaceous extracellular matrix	16	93	3.28	0.010482
CC	extracellular region	30	275	2.08	0.021195

Notes: ^a^category of GO term, ^b^number of DEGs enriched in a certain term, ^c^the number of all genes enriched in a certain term, ^d^degree of over-representation of DEGs compared with the background, ^e^the *p*-value adjusted using the Benjamini-Hochberg procedure.

Furthermore, we focused on ascorbate peroxidase (APX), a key enzyme for degrading reactive oxygen species (ROS) generated by symbionts. The expression level of APX homologues was measured by qPCR in the six experimental groups (electronic supplementary material, figure S2). The expression of APX decreased in both apo-symbiotic polyps, K10apo and M9apo, compared with K10ori and M9ori, respectively. Although M9exc recovered the expression level of APX almost the same as M9ori, K10exc did not recover its APX expression.

## 4. Discussion

Green hydra has a stable symbiotic relationship with *Chlorella*; however, it can survive even if symbiotic chlorellae are removed and can form a symbiotic relationship with symbiotic chlorellae of other green hydra strains [15,21,23–26]. However, it is unknown in detail how other strains of *Chlorella* affect the host. In this study, we established symbiont-exchanged hydras using genetically distant green hydra strains to determine the changes that occurred in their morphologies, proliferation rates and gene expression patterns. We found that the changes caused by harbouring non-native symbionts were remarkably different across the strains. In particular, the K10 strain was more sensitive to changes in its symbiotic state than the M9 strain.

### 4.1. Tolerance for and dependence on symbionts in green hydra

Our results showed that removal of symbiotic chlorellae reduced the asexual proliferation rate in both the K10 and M9 strains, and the extent of this reduction was clearly larger in the K10 strain than in the M9 strain, indicating that there was a difference in susceptibility to the absence of symbionts across strains of hydra (table 1). We expected the reduction in the asexual proliferation rate in apo-symbiotic polyps (K10apo or M9apo) to be recovered by reintroduction of original symbionts or those from other strains because a previous study reported that the exchange of symbiotic chlorellae among green hydra strains resulted in successful establishment of some new combinations of symbiont/host partnerships [21]. However, the reduction in the asexual proliferation rate could not be fully restored by reintroduction of the original symbiont (K10res and M9res). The apo-symbiotic strains have been kept for more than 10 years without symbiont, so it cannot be denied that some changes may have occurred during a long period of successive cultures. Therefore reintroduced strains, M9res and K10res, may have traits different from the original symbiotic ones. Furthermore, the asexual proliferation rate of symbiont-exchanged K10 polyps (K10exc) showed a much larger reduction compared with K10res, whereas the rate in M9exc was at the same level as that in M9res. In addition, we found no egg production in K10apo and K10exc. These results suggested that the K10 polyps were more dependent on symbionts than the M9 polyps or had low receptivity to changes in their symbiotic states.

Substantial morphological changes occurred in K10apo and K10exc but not in M9exc (table 2, figure 1). K10apo and K10exc showed a decrease in stenoteles, which play a main role in capturing prey, resulting in a decrease in predation success (table 2). In endodermal epithelial cells of host green hydra, each symbiotic chlorella is surrounded by the symbiosome membrane individually. In K10exc, however, some aggregations of chlorellae were found in a single symbiosome (figure 1*n*); this did not occur in M9exc. The symbiosome membrane was suggested to play an important role in the transportation of products involved in photosynthesis by algae to host cells in other symbiotic Cnidaria. Symbiosomes are normally located on the basal side (mesoglea side) of endodermal cells in native green hydra. This arrangement is convenient for symbiosomes to escape fusion with lysosomes. In the K10exc cells, however, the location of chlorellae in host cells changed to the upper side (gastric lumen side); this did not occur in M9exc cells. This result suggests that symbionts in K10exc have a greater risk of being digested by fusing with lysosomes.

These observations indicated that host/symbiont incompatibility has a pleiotropic effect on host polyps and suggested that such effects depend on differences in dependency on symbiotic chlorellae and tolerance to incompatible traits of non-native symbiotic chlorellae among the green hydra strains. The K10 strain is more dependent specifically on its original symbiotic chlorellae and has less tolerance to symbiont differences than the M9 polyps.

### 4.2. Gene expression was dramatically changed in K10apo and K10exc

The gene expression pattern was considerably changed in K10apo and K10exc compared with K10ori, in agreement with their significant morphological changes. In addition, these strains have similar gene expression patterns. Yuyama *et al*. [39] compared transcriptomes in juvenile corals of *Acropora tenuis* infected with different strains of zooxanthella species, *Symbiodinium goreaui* (clade C) and *S. trenchii* (clade D). They showed that the corals with a steady increase in the symbiont *S. trenchii* had more DEGs after infection compared with apo-symbiotic corals, whereas the corals with a slow increase in the symbiont *S. goreaui* showed unremarkable changes. GO enrichment analyses indicated that the presence of symbiotic algae has a great influence on coral metabolism in corals with *S. trenchii*, and such genes seem to play an important role in the establishment of endosymbiosis. K10exc did not seem to be able to establish an appropriate symbiosis with a new symbiont at the gene expression level because the gene expression patterns found in K10apo and K10exc were similar. A misrecognition of symbionts or incompatible interaction between polyps and symbionts may occur in K10exc polyps. Hamada *et al*. [15] conducted an experiment replacing the symbiont of *H. viridissima* A99 with *Chlorella variabilis* NC64A. The apo-symbiotic A99 polyps and the symbiont-exchanged A99 polyps whose symbiont was replaced with NC64A showed decreased proliferation rates similar to K10apo and K10exc in this study; however, the changes in gene expression found in the K10 and A99 strains were quite different. Furthermore, we found a few DEGs in M9apo compared with M9ori, although fewer replicates in our RNA-seq analysis (two biological replicates for each experimental group) might cause the reduced statistical power. The DEGs found in the M9exc polyps were essentially different from those found in the K10exc (table 4). These results suggest that the responses to a new symbiont at the gene expression level may differ among green hydra strains.

### 4.3. Cellular energy balance and response to reactive oxygen species in *H. viridissima* K10 and M9

In particular, the upregulation of genes with the GO term ‘translation’ and the downregulation of genes belonging to ‘electron transport chain complexes' were observed in both K10apo and K10exc (table 3). Although not significant in the enrichment analysis, some genes related to translation and respiration were downregulated in M9exc compared with M9ori (electronic supplementary material, table S6). Ishikawa *et al*. [19] showed that GO terms such as ‘translation’ and ‘respiratory chain’ were enriched in the upregulated genes in the apo-symbiotic M9 polyps. Interestingly, the expression patterns of the genes related to translation, respiratory chain and electron transport changed in host organisms of other endosymbiotic systems in the presence or absence of symbionts, although the direction of regulation is inconsistent. For example, genes involved in translation and the respiratory chain were upregulated only in the newly established symbiotic brown hydra (*Hydra vulgaris* 105G) but not in native symbiotic brown hydra (*H. vulgaris* J7) [40]. *Acropora tenuis* infected with *S. trenchii* (clade D) showed downregulation of genes involved in translation and upregulation of genes involved in electron transport compared with apo-symbiotic corals [39]. Moreover, in *P. bursaria*, which is a symbiotic protist, the genes related to the GO term ‘translation’ were upregulated in the apo-symbiotic cells [41].

Translation is one of the most ATP-consuming cellular processes [42,43] and is energy-balanced with the ATP-producing system and electron transport chain [44]. In marine invertebrates, the activity of the electron transport chain is regulated by oxygen partial pressure in the cell [45]. The removal of symbionts could cause the loss of oxygen production by photosynthesis, which may result in suppression of the function of the electron transport chain and change the energy balance in the cells. Although we did not examine the photosynthesis activities in K10exc, the non-native symbiont could not produce oxygen because of a lack of proper photosynthesis processes, and the function of the electron transport chain might be suppressed. For one thing, some aggregations of chlorellae were found in a single symbiosome of K10exc (figure 1*n*). It indicated that at least the division of the non-native symbiotic chlorellae was not coordinated with symbiosome formation. It may inhibit normal nutrient exchange between the host and the symbiont necessary for photosynthesis.

Furthermore, ATP production by the respiratory chain and photosynthesis generates ROS [46,47]. While polyps receive some benefits, such as photosynthetic products, from the symbiont, they are exposed to ROS damage. In coral-*Symbiodinium* symbiosis, ROS generated by the symbiont at a high temperature can cause coral bleaching, which expels the symbiont necessary for coral survival from the coral cells due to cellular damage by ROS [48,49]. APX is one of the major enzymes that acts in the ROS degradation process. We found that one APX homologue was downregulated in K10apo and K10exc, while it was downregulated only in apo-symbiotic polyps in the M9 strain (electronic supplementary material, figure S2). APX is an enzyme that degrades H_2_O_2_ to H_2_O in plants [50]. Habetha and Bosch [22] found that the gene coding for a plant-related ascorbate peroxidase in green hydra (HvAPX1) was only expressed in symbiotic hydra. To date, this enzyme has not been found in other animals [22]. Our results agree with this previous study, except that APX was downregulated in K10exc. The production of ROS and its degradation by peroxidase can be balanced in hydra cells with native symbiotic *Chlorella*. In the K10 strain, the expression of APX also decreased when native symbionts were removed or replaced, suggesting that the production of ROS by the electron transport chain and by photosynthesis decreased in the host cell (electronic supplementary material, figure S2). If the replaced symbiont has a normal photosynthesis activity in K10, it could cause ROS degradation failures and damage to host cells. On the other hand, replaced symbiont seems to have nearly normal level of photosynthesis activity in M9 because APX was expressed at near normal levels in M9exc.

The expression changes in the genes for intracellular energy balance and APX in apo-symbiotic and symbiont-exchanged hydras indicated that host cells strictly regulate their energy production and consumption by their symbiotic state with a proper interaction with symbionts. If this mechanism does not work well, pleiotropic effects could appear on host hydras, such as a reduction in proliferation and impaired changes in morphologies.

### 4.4. Evolution of symbiosis systems in *Hydra*

Our previous study suggested that a balanced cellular metabolic system would be a key factor for stabilizing and adapting to symbiosis at the early stage of symbiosis evolution in the symbiotic brown hydra, *H. vulgaris* [40]. Interestingly, the K10exc polyps showed drastic changes in the expression pattern of genes involved in translation and the respiratory chain, like a newly established symbiotic brown hydra (*H. vulgaris* 105G), suggesting a cellular metabolic system in the K10exc cells would be unbalanced (table 3). An incompatibility of host/symbiont combination may result in a reversion to an unstable state as in the early stage of symbiosis evolution. Furthermore, such a mechanism would be highly sophisticated and diversified through the lineage-specific coevolution of *H. viridissima* and *Chlorella* with a long evolutionary history that would be more efficient and adaptive to various environments. At the same time, it is expected that the evolution of a more strict recognition system and synchronization of life cycles would occur between host and symbiont, at least in some strains, which cause a difference in response to a new (non-native) symbiont among strains. Further comparative analysis with omics techniques (proteome, metabolome) between the 105G and native symbiotic strains (J7) in *H. vulgaris* and between the K10 and M9 strains in *H. viridissima* will provide an overview of the evolution of the symbiotic system in *Hydra*.

### 4.5. Conclusion

The symbiont removal and replacement experiments and the differential gene expression analyses in the K10 and M9 strains provided insights into the effects of symbionts on hosts and their specificity. In the K10 strain, the general trend in morphological, behavioural and gene expression changes was similar with the removal and replacement of the symbionts. By contrast, no such trend was observed in the M9 strain. Significantly, the gene expression changes related to translation and the electron transport chain were notable when symbionts were removed or exchanged in the K10 strain. Such changes were also found in other cnidaria species harbouring endosymbionts. This result suggests that dynamic changes in cellular metabolism and cellular energy balance are necessary to establish and maintain symbiosis in green hydra. Violation of such mechanisms may cause pleiotropic effects on host morphologies or behaviours. Comparison of the gene expression patterns by symbiont removal and exchange experiments enables us to determine what kind of genes are responsible for establishing symbiosis associations and specificities. These results will also provide insight into the evolutionary process and origins of symbiosis. However, comprehensive gene expression comparisons alone could not clarify the link between morphological or behavioural changes and the gene involved. Gene knockout experiments or *in situ* hybridization with symbiont exchange experiments will be necessary to elucidate the functions of the genes related to endosymbiosis. In addition, gene expression changes in symbiont algae are also necessary to elucidate changes in cellular metabolism and cellular energy balance in endosymbiotic cells.

## Data Availability

The sequence data were deposited in the DDBJ/EMBL/Genbank under the accession numbers: SRR21134050–SRR21134067. The assembled contig sequences and the clustering information are available from the following https://doi.org/10.5061/dryad.2fqz612rq [51]. The data are provided in electronic supplementary material [52].
